# Role of gut microbiome in pathogenesis and treatment of diseases: Multidisciplinary experts’ opinion of the Asian Medical Experts Academy (AMEA)

**DOI:** 10.1080/29933935.2025.2558575

**Published:** 2025-09-29

**Authors:** Raphaela I. Lau, Martin C. S. Wong, Louis H. S. Lau, Alexander Y. L. Lau, Ivan W. C. Mak, Oswens S. H. Lo, Norman N. Chan, David L. K. Dai, Kwan C. Hau

**Affiliations:** a Microbiota I-Center (MagIC), Hong Kong SAR, China; b Department of Medicine and Therapeutics, Faculty of Medicine, The Chinese University of Hong Kong, Hong Kong SAR, China; c Jockey Club School of Public Health and Primary Care, Faculty of Medicine, The Chinese University of Hong Kong, Hong Kong SAR, China; d Department of Psychiatry, Faculty of Medicine, The Chinese University of Hong Kong, Hong Kong SAR, China; e Asian Medical Experts Academy, Asian Medical Experts Academy Hong Kong, Hong Kong SAR, China

**Keywords:** Gut microbiome, probiotics, prebiotics, synbiotics, microbiome-based therapeutics, clinical guidelines, clinical application, experts’ opinion

## Abstract

Emerging evidence suggests the role of the gut microbiome in the health and diseases of multiple organs and systems. In the past decade, an increasing trend in the use of microbiome-based therapeutics (e.g., probiotics, prebiotics, synbiotics) has been observed in Asia and globally. However, local and global clinical guidelines on the use of microbiome-based therapeutics are limited. A multidisciplinary working group has been established to foster communication between experts from diverse medical specialties on the clinical application of microbiome-based therapeutics. Through conducting an extensive review on current evidence on the importance of the gut microbiome and the potential use of microbiome-based therapeutics in health and diseases, the experts’ working group identified the unmet needs related to the use of microbiome-based therapeutics in the clinical settings in Asia and global contexts. Thirteen position statements were developed, including eight statements focusing on the role of the gut microbiome in health and disease pathogenesis, as well as six statements focusing on the potential clinical applications. A list of potential indications for microbiome-based therapeutics was also proposed based on current evidence and clinical experience. This paper is intended to serve as a reference that assists healthcare professionals in improving care for patients using microbiome-based therapeutics in Asia and globally.

## Introduction

The gut microbiota, which consists of trillions of microorganisms (i.e., bacteria, viruses, fungi, and archaea), plays crucial roles in health and diseases of multiple organs and systems, including the digestive system, immune system, mental health, skin health, endocrine function, and even infectious diseases like coronavirus disease 2019 (COVID-19).^1–9^ Rapid advancements in high-throughput sequencing technology and multi-omic analysis have transformed our understanding of the microbial composition and dynamics in the gut and their extensive impact on human health.^10–12^ Through observational studies, researchers have been able to compare and analyze the microbial profiles of healthy individuals with those suffering from a disease, revealing key microbial signatures that are significantly associated with different clinical conditions.^13,14^ Functional analyses have also shed light on the underlying mechanisms and pathways through which the gut microbiota influences pathophysiological processes.^5,10,11^ Furthermore, emerging animal studies have begun to demonstrate the causal links between gut microbiota and the development of diseases *in vivo*.^15–19^ These data act as a powerful reference that facilitates the development of targeted therapeutics for disease prevention and management.

Modulation of gut microbiota has emerged as a novel therapeutic approach for different conditions.^20^ Diet plays a fundamental role in shaping the gut microbiome composition and diversity.^21,22^ Apart from dietary modulation, other microbiome-based interventions such as probiotics, prebiotics, and synbiotics have garnered significant interest in recent years for their potential to alter the gut microbiome and impact the pathophysiology of various organs and systems.^23^ Probiotics, which are live microorganisms providing health benefits when consumed in adequate quantities, have attracted significant attention in the past decade.^24^ Prebiotics, which are substrates promoting the growth of host microorganisms,^25^ as well as synbiotics, which combine probiotics and prebiotics for enhanced effects,^26^ offer additional avenues for gut microbiome modulation. Key probiotic strains that are seen in most over-the-counter formulations, including *Bifidobacterium* and *Lactobacillus*, have been extensively studied in clinical and animal studies for their health-promoting and immunomodulatory potential across various organ systems.^27,28^

The use of probiotics and synbiotics is widespread in Asia, and globally, reflecting a worldwide trend of increased awareness and incorporation of microbiome-based supplements into routine health regimens.^29,30^ Nevertheless, the lack of comprehensive clinical guidelines poses a significant challenge for both healthcare professionals and consumers. Current guidelines, which are often published by professional bodies of a particular medical specialty, focus primarily on respective diseases of the specialty rather than providing holistic recommendations for general probiotic use. In addition, the efficacy and safety data on probiotics and synbiotics in different diseases and conditions are scattered across numerous studies, complicating the decision-making for healthcare professionals, who may struggle with uncertainties regarding the appropriate indications, dosage and formulation for probiotics and synbiotics. The rapidly evolving landscape of microbiome research has also been a common challenge for front-line healthcare professionals to keep track of the cutting-edge evidence on the role of the gut microbiome in pathogenesis and treatment of diseases. There is an urgent need for an up-to-date and comprehensive guideline on the clinical application of probiotics and synbiotics in Asia and globally.

Recognizing the unmet need for evidence-based guidance on probiotic use, a multidisciplinary working group representing the Asian Medical Experts Academy (AMEA) has been established in August 2024 to foster communication between experts from diverse medical specialties on the clinical application of microbiome-based therapeutics based on state-of-the-art scientific evidence and clinical experience. This working group aimed (i) to identify and address the unmet needs related to the use of microbiome-based therapeutics in the clinical settings; (ii) to review current evidence on the importance of gut microbiome and the potential use of microbiome-based therapeutics in health and diseases; and (iii) to bring forward potential clinical application of microbiome-based therapeutics in Asia and global contexts. In this paper, we discuss the position statements developed based on the experts’ opinion consolidated within the working group (Box 1), covering the role of gut microbiome in health and disease pathogenesis (statements 1–8), as well as the potential clinical applications (statements 9–13). A list of potential indications for microbiome-based therapeutics was also proposed based on current evidence and clinical experience (Box 2).

Box 1. Panel statements on the role of gut microbiome in pathogenesis and treatment of diseases.

**Role of gut microbiome in health and disease pathogenesis** Statement 1: Gut dysbiosis is a common condition characterized by an imbalance of gut microbiota composition that is linked to diet, diseases and use of medication. Statement 2: The gut microbiota plays profound roles in health and pathogenesis of diseases in multiple organs and systems, such as cancer, gastrointestinal, neuropsychiatric, skin, endocrine, cardiovascular and respiratory conditions. Existing evidence suggests significant differences in gut microbiota composition and functions between those with a disease and healthy controls. Statement 3: Causal relationships between the gut microbiota and multiple diseases have been shown by emerging studies adopting animal models and the Mendelian randomization method. Statement 4: Six important features of gut dysbiosis include (i) an increase in pathogenic microorganisms; (ii) an increase in pathogenic microbial metabolites or products; (ii) an increase in intestinal permeability and inflammation; (iv) a decrease in beneficial microorganisms; (v) a decrease in beneficial microbial metabolites or products; and (vi) a decrease in microbial diversity. Statement 5: Impairment of intestinal barrier/increase in intestinal permeability (“leaky gut”) is central to the pathogenesis of different diseases. Gut dysbiosis could cause damage to the intestinal barrier, which allows the passage of toxins or disease-causing microbial components and metabolites into the bloodstream, thereby affecting different parts of the human body via various gut-organ axes, such as gut-skin axis, gut-lung axis and gut-brain axis. Statement 6: Increased fecal lipopolysaccharides (LPS), a component of the gram-negative bacterial outer membrane with pro-inflammatory properties, is a key contributing factor in the initiation and progression of low-grade systemic inflammation, which is observed across multiple diseases. Statement 7: Short-chain fatty acids (SCFAs) produced by the gut microbiota play beneficial roles in health. SCFAs could potentially improve intestinal barrier functions, regulate innate and adaptive immunity, and inhibit tumorigenesis. Statement 8: Gut dysbiosis could affect tumorigenesis across different cancer types. Some pathogenic gut bacteria could stimulate a chronic inflammatory state that is associated with carcinogenesis; some bacteria could alter intracellular signaling pathways that regulate cell growth and proliferation. **Potential clinical applications of microbiome-based therapeutics** Statement 9: Modulation of gut microbiota using probiotics or synbiotics is recommended as a complementary strategy to improve intestinal barrier functions and manage a wide array of diseases along with standard care, such as cancer, gastrointestinal, neuropsychiatric, skin, endocrine, and respiratory conditions. Statement 10: Supplementation of probiotics or synbiotics may offer health benefits such as altering host immune response, lowering the growth of pathogenic microorganisms and enhancing the microbial balance, when used in a continuous manner. Regular maintenance of gut microbial balance is recommended for both healthy individuals and those with a disease. Statement 11: When selecting synbiotics, the probiotic strains should be chosen based on the health benefits that it may provide to the individual as guided by both local and global scientific and clinical evidence, while prebiotics or other substrates are used in combination to promote the growth and activities of beneficial members of the gut microbiota and provide a health benefit. Statement 12: Microbial signatures of health and diseases are heterogeneous across geographical locations. It is recommended to take local clinical and microbiome data into consideration when determining the most optimal synbiotics for the local population. Statement 13: It is a common misbelief that higher doses and more probiotic bacteria assure greater health benefits. The optimal combination of relative proportions of specific bacteria, as guided by available scientific and clinical evidence, is the most important factor governing clinical outcomes.

Box 2. Potential indications for microbiome-based therapeutics.

**Gastrointestinal conditions**: irritable bowel syndrome, inflammatory bowel disease, constipation, diarrhea, acute gastroenteritis, GI cancer, colorectal adenoma **Skin conditions**: atopic dermatitis, acne vulgaris, alopecia, contact dermatitis **Geriatric conditions**: Alzheimer’s disease, mild cognitive impairment, hypertension, coronary heart disease, chronic kidney disease **Psychiatric conditions**: depression, anxiety, autism spectrum disorder, attention deficit/hyperactivity disorder, bipolar disorder, eating disorders **Neurological conditions**: post-stroke management, Parkinson’s disease, multiple sclerosis **Endocrine conditions**: hypothyroidism, hyperthyroidism, thyroid cancer, thyroiditis, polycystic ovary syndrome **Respiratory conditions**: coronavirus disease 2019, seasonal influenza, allergic rhinitis, asthma, chronic obstructive pulmonary disease **Metabolic conditions**: metabolic syndrome, diabetes mellitus, nonalcoholic fatty liver disease, obesity, dyslipidaemia **Other conditions related to gut dysbiosis**: post-antibiotics, post-infection conditions, autoimmune conditions, systemic inflammatory disease, allergy, cancer **Health maintenance**: immunity boosting, disease prophylaxis

## Methods

In August 2024, a multidisciplinary working group representing the Asian Medical Experts Academy (AMEA) was established to develop the guideline statements. Selection of working group members was based on expertise in microbiome-based therapeutics through research track record and/or participation as key opinion leaders in the field. A total of eight medical specialists were included into the working group, including a gastroenterologist (LHSL), a neurologist (AYLL), a psychiatrist (IWCM), a dermatologist (KCH), an endocrinologist (NNC), a geriatrician (DLKD), a general surgeon (OSHL), and a family medicine specialist (MCSW), to ensure a diverse range of expertise across specialties.

Literature search on the role of the gut microbiome in pathogenesis and treatment of diseases was performed using PubMed/MEDLINE and Google Scholar databases, with keywords including “gut microbiome” or “gut microbiota” or “probiotics” or “prebiotics” or “synbiotics.” Global and local research findings published in the English language up to 20 November 2024 were reviewed. Publications were screened based on title and abstract; studies were excluded if the content was regarded as irrelevant. Relevant clinical trials conducted in Hong Kong up to 20 November 2024 were also identified on ClinicalTrials.gov. A set of initial statements were drafted based on published observational studies, clinical trials, *in vitro* and *in vivo* studies, case reports, systematic reviews, and meta-analyses, after which all group members were asked to provide their feedback independently. A collection of selected literature was made available to all panel members.

A face-to-face meeting of the working group was held on 7 December 2024 in Hong Kong, China. During the meeting, the initial statements were shown, allowing the panel members to discuss and suggest modifications to the statements based on current literature and clinical experience. Panel members discussed statements with divergent views on the role of the gut microbiome in health and disease pathogenesis, as well as the potential clinical applications. The statements were further revised according to the discussion among all members. Literature to support or refute the revised statements was gathered based on global and local research findings.

A post-meeting survey was conducted with an internet-based platform. Panel members were asked to rank each statement on a 5-point Likert scale (A = “There is good evidence to support the statement”; B = “There is fair evidence to support the statement”; C = “There is poor evidence to support the statement but recommendation made on other grounds”; D = “There is fair evidence to refute the statement”; E = “There is good evidence to refute the statement”). Agreement (A or B on the Likert scale) by ≥75% of the panel members was defined *a priori* as consensus. Level of agreement for each statement in the post-meeting survey was expressed as the proportion of votes at each point on the Likert scale. Consensus was reached among all panel members on the final statements. The statement development process included face-to-face meetings, video conferences, online discussions, and voting among panel members between 2 September 2024 and 19 March 2025.

## Results

### Role of gut microbiome in health and disease pathogenesis

Statement 1: Gut dysbiosis is a common condition characterized by an imbalance of gut microbiota composition that is linked to diet, disease, and use of medication. *(A: 87.5%; B: 12.5%; C: 0%; D: 0%; E: 0%).*

**Figure 1.**
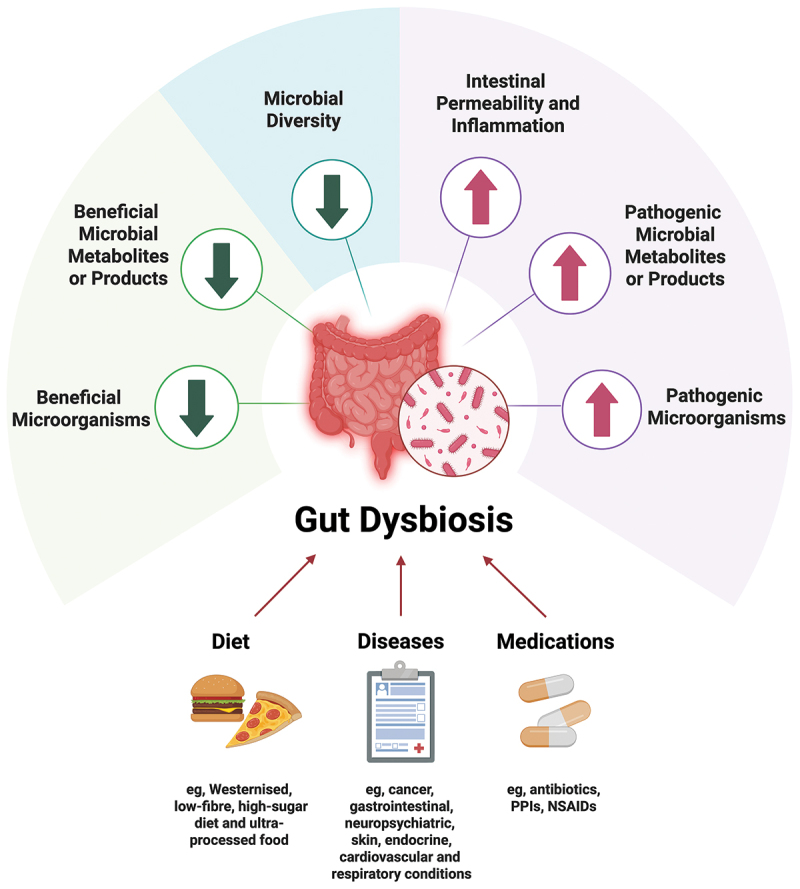
Gut dysbiosis and its key features. The gut microbiota plays profound roles in health and pathogenesis of diseases in multiple organs and systems, such as cancer, gastrointestinal, neuropsychiatric, skin, endocrine, cardiovascular and respiratory conditions. Gut dysbiosis is a common condition characterized by an imbalance of gut microbiota composition that is linked to diet, diseases and use of medication. Six important features of gut dysbiosis include an increase in pathogenic microorganisms; an increase in pathogenic microbial metabolites or products; an increase in intestinal permeability and inflammation; a decrease in beneficial microorganisms; a decrease in beneficial microbial metabolites or products; and a decrease in microbial diversity. Figure created with BioRender.com.

Gut dysbiosis is characterized by changes in microbial composition, diversity, and function of the gastrointestinal (GI) tract, marked by an increase in the relative abundance of potentially pathogenic microbes and a decrease in the relative abundance of potentially beneficial microbes (Figure 1).^1–9^ The imbalance may lead to alterations in the interactions between the gut microbiota and the host, potentially affecting host health and contributing to the development or progression of diseases or conditions. A growing body of evidence has shown that gut dysbiosis is prevalent in individuals with a range of health conditions, for instance, inflammatory bowel disease (IBD), irritable bowel syndrome (IBS), neuropsychiatric disorders, metabolic disorders, and skin conditions.^1–9,15,31–35^ Gastrointestinal symptoms, which are commonly linked to gut dysbiosis, are also observed clinically in non-GI conditions, such as mood disorders (e.g., depression, anxiety), thyroid disorders (e.g., hyperthyroidism, hypothyroidism), neurological disorders (e.g., Parkinson’s disease), and infectious diseases (e.g., coronavirus disease 2019, seasonal influenza).^36–40^ Dietary factors, particularly the increased consumption of Westernized, low-fiber, high-sugar diet, and ultra-processed food, may contribute to dysbiosis.^21,22^ Antibiotic use is also a common factor in severely disrupting the gut microbiome.^41^ Several medications, such as proton pump inhibitors (PPIs) and non-steroidal anti-inflammatory drugs (NSAIDs), have also been associated with alterations in gut microbiome composition, potentially leading to dysbiosis and gastrointestinal side effects.^42–44^ Overall, gut dysbiosis is understood to be a condition that may affect a significant portion of the global population given the influence of diet, diseases, and use of medications.

Statement 2: The gut microbiota plays profound roles in the health and pathogenesis of diseases in multiple organs and systems, such as cancer, gastrointestinal, neuropsychiatric, skin, endocrine, cardiovascular, and respiratory conditions. Existing evidence suggests significant differences in gut microbiota composition and functions between those with the disease and healthy controls. *(A: 75.0%; B: 25.0%; C: 0%; D: 0%; E: 0%).*

**Figure 2.**
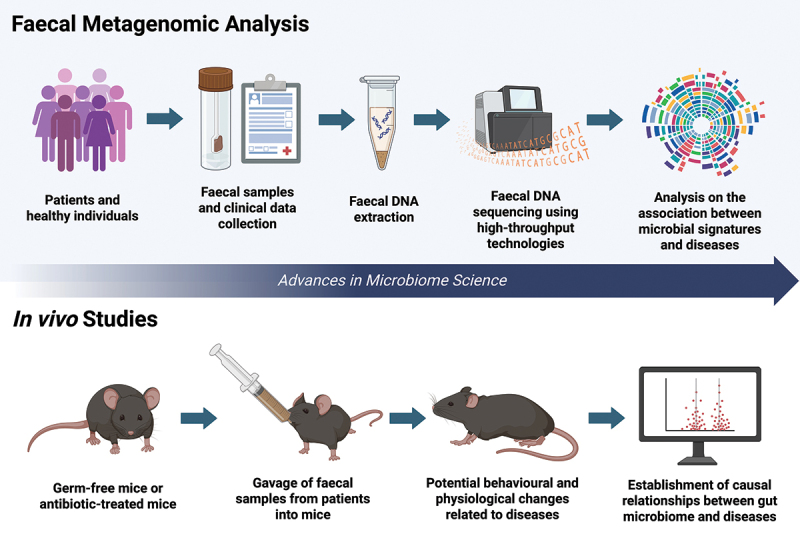
Advances in microbiome science. Rapidly emerging research using fecal metagenomic analysis have shed light on the important role of gut microbiome in health and disease pathogenesis. By collecting fecal samples, fecal metagenomics examine the genetic material of microbial communities in the gut, providing insights into differences in gut microbiota composition, diversity, and functions between healthy individuals and those suffering from a disease. Emerging studies using animal models have enabled researchers to further demonstrate causal relationships, offering strong evidence that the gut dysbiosis is an active contributor to disease pathogenesis. In vivo models, particularly germ-free (GF) mice that are raised in sterile environments or antibiotic-treated mice, may serve as robust tools for studying the causal relationship. Through gavaging GF or antibiotic-treated mice with fecal samples collected from healthy individuals and those with a disease, researchers could compare the impact of microbial composition on host physiology. Figure created with BioRender.com.

Rapidly emerging research using fecal metagenomic analysis has shed light on the important role of gut microbiome in health and disease pathogenesis (Figure 2).^1–9,15,31–35^ By collecting fecal samples, fecal metagenomics examine the genetic material of microbial communities in the gut, providing insights into differences in gut microbiota composition, diversity, and functions between healthy individuals and those suffering from a disease.^10–12^ The process begins with the extraction of deoxyribonucleic acid (DNA), followed by library preparation, sequencing using high-throughput technologies, as well as downstream data processing and analysis.^10–12^ While taxonomic profiling could aid in the classification of microbial species, functional annotation could aid in the prediction of metabolic functions.^10–12^ Diversity analysis measures within-sample (i.e., alpha diversity) and between-sample (i.e., beta diversity) variations.^10–12^ To date, fecal metagenomic data have revealed significant gut dysbiosis in numerous diseases. Examples discussed within the working group include gastrointestinal conditions (e.g., IBS, IBD, constipation, diarrhea, acute gastroenteritis, GI cancer, colorectal adenoma),^31,32,45–47^ skin conditions (e.g., atopic dermatitis, acne vulgaris, alopecia, contact dermatitis),^8^ geriatric conditions (e.g., Alzheimer’s disease, mild cognitive impairment, hypertension, coronary heart disease, chronic kidney disease),^4,6,35,48,49^ psychiatric conditions (e.g., depression, anxiety, autism spectrum disorder, attention deficit/hyperactivity disorder, bipolar disorder, eating disorders),^4,33,50–53^ neurological conditions (e.g., stroke, Parkinson’s disease, multiple sclerosis),^4,54–56^ endocrine conditions (e.g., hypothyroidism, hyperthyroidism, thyroid cancer, thyroiditis, polycystic ovary syndrome),^57,58^ respiratory conditions (e.g., COVID-19, seasonal influenza, allergic rhinitis, asthma, chronic obstructive pulmonary disease),^59–62^ metabolic conditions (e.g., metabolic syndrome, diabetes mellitus, nonalcoholic fatty liver disease, obesity, dyslipidaemia),^3,34,63–65^ and others (e.g., autoimmune conditions, systemic inflammatory disease, allergy, cancer).^60,66–68^

Statement 3: Causal relationships between the gut microbiota and multiple diseases have been shown by emerging studies adopting animal models and the Mendelian randomization method. *(A: 37.5%; B: 62.5%; C: 0%; D: 0%; E: 0%).*

While observational studies have revealed the intriguing associations between the gut microbiota and a wide range of diseases, a majority of studies were only capable of establishing associations rather than causation. Emerging studies using animal models and Mendelian randomization (MR) have enabled researchers to further demonstrate causal relationships, offering strong evidence that the gut dysbiosis is an active contributor to disease pathogenesis.^15–19,69–76^ *In vivo* models, particularly germ-free (GF) mice that are raised in sterile environments or antibiotic-treated mice, may serve as robust tools for studying the causal relationship (Figure 2).^77,78^ Through gavaging GF or antibiotic-treated mice with fecal samples collected from healthy individuals and those with a disease, researchers could compare the impact of microbial composition on host physiology.^77,78^ For instance, studies have shown that transplanting gut microbiota from IBS patients to GF mice could induce changes in gastrointestinal transit, gut barrier functions, innate immunity, as well as behavior in the recipient mice.^18^ GF mice colonized with gut microbiota from colorectal cancer patients also showed elevated proportions of proliferating cells in the colon.^17^ These results provided evidence that the gut microbiota could induce disease states, independent of potential confounding factors. Mendelian randomization (MR) is another powerful tool that leverages genetic variants to demonstrate whether an observational association between certain microbial signatures and disease outcomes aligns with a causal relationship.^79^ MR studies have identified causal links between gut microbiota and various diseases such as cancers, gastrointestinal diseases, psychiatric disorders, autoimmune conditions, cardiovascular diseases, chronic respiratory diseases, and allergic diseases.^69–76^

Statement 4: Six important features of gut dysbiosis include (i) an increase in pathogenic microorganisms; (ii) an increase in pathogenic microbial metabolites or products; (ii) an increase in intestinal permeability and inflammation; (iv) a decrease in beneficial microorganisms; (v) a decrease in beneficial microbial metabolites or products; and (vi) a decrease in microbial diversity. *(A: 75.0%; B: 25.0%; C: 0%; D: 0%; E: 0%).*

Six key features of gut dysbiosis are commonly observed in disease states and are interconnected with one another (Figure 1). The first feature is the increase in pathogenic microorganisms. Under normal circumstances, the gut microbial community coexists in a symbiotic relationship.^80^ Factors such as antibiotic use and poor diet could disrupt the balance, leading to an increase in the relative abundance of harmful bacteria.^21,41^ The second feature is the increase in pathogenic microbial metabolites or products, for instance, lipopolysaccharides (LPS), a bacterial endotoxin that could trigger inflammation when it crosses the gut barrier and enters the bloodstream, leading to both local and systemic effects.^81^ The third feature is the increase in intestinal permeability and inflammation.^82,83^ The gut epithelium serves as an important barrier that prevents the translocation of pathogenic bacteria and their metabolites from the gut into the bloodstream.^82,83^ Some pathogenic bacteria are able to disrupt the barrier, resulting in increased intestinal permeability.^82,83^ The fourth feature is the decrease in beneficial microorganisms. Beneficial bacteria play important roles in maintaining gut homeostasis by competing with pathogenic bacteria for nutrients, and modulating the immune response.^80^ Factors including antibiotic use, diet high in ultra-processed food, emulsifier, and preservatives and low in dietary fiber, may affect the growth of beneficial bacteria.^21,22,41^ The fifth feature is the decrease in beneficial microbial metabolites or products. Beneficial bacteria produce a variety of metabolites that are essential for maintaining gut and overall health, such as short-chain fatty acids (SCFAs).^84^ The final feature is the decrease in microbial diversity. A healthy and balanced gut ecosystem is characterized by a high degree of diversity with different bacteria performing different functions.^80^ In a state of dysbiosis, the diversity of the gut microbiota is often reduced, leading to a loss of vital functions, such as breaking down complex carbohydrates, producing vitamins, and modulating the immunity.^1,2^

**Statement 5**: Impairment of the intestinal barrier/increase in intestinal permeability (“leaky gut”) is central to the pathogenesis of different diseases. Gut dysbiosis could cause damage to the intestinal barrier, which allows the passage of toxins or disease-causing microbial components and metabolites into the bloodstream, thereby affecting different parts of the human body via various gut-organ axes, such as gut-skin axis, gut-lung axis, and gut-brain axis. *(A: 62.5%; B: 25.0%; C: 12.5%; D: 0%; E: 0%).*

**Figure 3.**
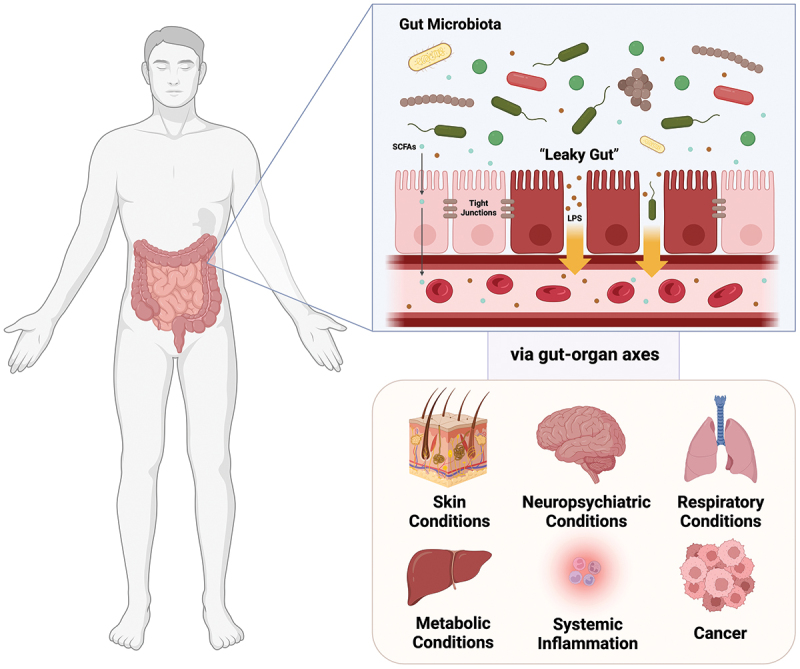
Concept of “leaky gut” and its associations with disease pathogenesis. The intestinal barrier is an intricate and dynamic structure that serves as the first line of defense against pathogens and toxins. Impairment of intestinal barrier/increase in intestinal permeability (“leaky gut”) is central to the pathogenesis of different diseases. Gut dysbiosis could cause damage to the intestinal barrier. While beneficial bacteria contribute to maintaining tight junction functions by producing short-chain fatty acids; pathogenic bacteria produce toxins and metabolites that may damage the intestinal epithelium and disrupt tight junctions. “leaky gut” could potentially trigger systemic inflammation and affect multiple organs and systems through various gut-organ axes, such as gut-skin axis, gut-lung axis and gut-brain axis. Figure created with BioRender.com.

The concept of “leaky gut” has gained a considerable amount of attention in recent years, as more studies reveal the local and systemic health consequences of an increased intestinal permeability, driven by gut dysbiosis (Figure 3).^5,82,83^ The intestinal barrier is an intricate and dynamic structure that serves as the first line of defense against pathogens and toxins.^5,82,83^ Tight junctions are protein complexes that form a critical component of the intestinal barrier and protect its integrity through preventing the translocation of harmful substances into the bloodstream.^85^ While beneficial bacteria contribute to maintaining tight junction functions by producing SCFAs; pathogenic bacteria produce toxins and metabolites that may damage the intestinal epithelium and disrupt tight junctions.^5,86^ “leaky gut” could potentially trigger systemic inflammation and affect multiple organs and systems through various gut-organ axes.^87^ Gut-organ axes highlight the bidirectional communications through which the gut microbiota and its metabolites influence the function of distant organs, and vice versa.^88^ One such axis is the “gut-skin axis,” which describes the relationship between the gut microbiota and skin health.^8^ Systemic inflammation induced by “leaky gut” may affect the skin microbiota and impair skin barrier function, resulting in the development or exacerbation of skin conditions.^8^ Another important axis is the “gut-lung axis,” through which the gut microbiota influences lung immunity, function, and inflammatory response.^89^ Last but not least, “gut-brain axis” is one of the most well-studied gut-organ axes to date.^4^ Increased intestinal permeability has been linked to systemic inflammation and the subsequent activation of immune cells that contribute to neuroinflammation.^6^

Statement 6: Increased fecal lipopolysaccharides (LPS), a component of the gram-negative bacterial outer membrane with pro-inflammatory properties, is a key contributing factor in the initiation and progression of low-grade systemic inflammation, which is observed across multiple diseases. *(A: 50.0%; B: 37.5%; C: 12.5%; D: 0%; E: 0%).*

Lipopolysaccharides (LPS) is a major structural component of the outer membrane of gram-negative bacteria, such as *Escherichia coli*.^81^ Under normal conditions, the intestinal barrier prevents the translocation of LPS from the gut into the systemic circulation.^82,83,86^ In case of “leaky gut,” LPS translocation is capable of inducing cascades of immune responses that contribute to low-grade systemic inflammation, a state that is increasingly recognized as a central and common mechanism underlying disease pathogenesis.^5,82,83,86^ LPS has been linked to many health conditions, including but not limited to gastrointestinal conditions (e.g., inflammatory bowel disease, irritable bowel syndrome), metabolic conditions (e.g., diabetes mellitus, obesity), neurodegenerative diseases (e.g., Alzheimer’s disease, Parkinson’s disease), and cancers.^90–95^ The process by which LPS induces systemic inflammation begins with its recognition by pattern recognition receptors (PRRs) (e.g., toll-like receptors) expressed on immune cells, followed by activation of signaling cascades leading to the production and release of pro-inflammatory cytokines (e.g., tumor necrosis factor-α, interleukin-1β, interleukin-6).^81–83,86,96^ This would result in a state of chronic, low-grade systemic inflammation with elevated levels of circulating pro-inflammatory cytokines and immune cell activation, damaging tissues and organs in a prolonged manner, and causing disease states.^81^

Statement 7 Short-chain fatty acids (SCFAs) produced by the gut microbiota play beneficial roles in health. SCFAs could potentially improve intestinal barrier functions, regulate innate and adaptive immunity, and inhibit tumorigenesis. *(A: 62.5%; B: 37.5%; C: 0%; D: 0%; E: 0%).*

Short-chain fatty acids (SCFAs), including butyrate, acetate, and propionate, are beneficial metabolites produced by the gut microbiota through fermentation of dietary fiber.^84^ SCFAs have gained significant attention in recent years due to their roles in gut and overall health, particularly the ability to enhance and maintain gut barrier functions.^84,97^ Butyrate is an SCFA that serves as a key energy source for colon epithelial cells and promotes their proliferation and differentiation, which is key to the maintenance of a robust barrier.^98^ SCFAs may also enhance the expression of tight junction proteins that prevent the translocation of pathogenic bacteria and metabolites into the bloodstream and the subsequent inflammatory response.^99^ Furthermore, SCFAs have been shown to regulate mucus production of goblet cells in the gut, enhancing protection for barrier integrity.^100^ Beyond their role in intestinal barrier functions, SCFAs are actively involved in immunomodulation through regulating the inhibition of pro-inflammatory cytokines (e.g., interleukin-6), the promotion of anti-inflammatory cytokines (e.g., interleukin-10), as well as the functions of dendritic cells, macrophages, and neutrophils.^101^ SCFAs are also able to exert systemic effects on various organs and physiological processes. For instance, SCFAs have been implicated in lipid and glucose metabolism, neuropsychiatric functions, and even suppression of tumor cell proliferation.^102–104^

Statement 8: Gut dysbiosis could affect tumorigenesis across different cancer types. Some pathogenic gut bacteria could stimulate a chronic inflammatory state that is associated with carcinogenesis; some bacteria could alter intracellular signaling pathways that regulate cell growth and proliferation. *(A: 25.0%; B: 50.0%; C: 25.0%; D: 0%; E: 0%).*

Numerous studies have shed light on the association between gut microbiota and tumorigenesis across cancer types, such as colorectal cancer (CRC), hepatocellular carcinoma (HCC), gastric cancer, and breast cancer.^68^ Gut dysbiosis has been implicated in the development and progression of cancer through various mechanisms. One potential mechanism is the promotion of chronic inflammation, which is known to alter intercellular communications in the tumor microenvironment that promotes cancer development.^68,105^ Some pathogenic gut bacteria (e.g., *Fusobacterium nucleatum*) have been implicated in cancer, especially CRC, by stimulating a chronic inflammatory state.^106–108^ *F. nucleatum* is closely linked to the activation of the nuclear factor kappa-light-chain-enhancer of activated B cells (NF-κB) signaling pathway, which promotes tumor cell proliferation, inhibits cell apoptosis, and regulates angiogenesis.^107,108^ In addition, the gut microbiota may influence host cell signaling via the production of metabolites such as secondary bile acids and short-chain fatty acids (SCFAs).^109,110^ A secondary bile acid, deoxycholic acid (DCA), is implicated in the development of CRC and is linked to the activation of the epidermal growth factor receptor (EGFR) signaling pathway, which promotes tumor proliferation and survival.^109^ In contrast, SCFAs produced by beneficial gut bacteria possess anti-tumorigenic potential in modulating gene expression, including the upregulation of tumor suppressor genes.^110^ Furthermore, gut dysbiosis may promote tumorigenesis through immunomodulation and create an immunosuppressive tumor microenvironment. For instance, *F. nucleatum* was shown to inhibit natural killer cells and cytotoxic T cells, which are key players in recognizing and destroying cancer cells.^108,111^ *F. nucleatum* promotes the recruitment of myeloid-derived suppressor cells (MDSCs), which suppress T cell activity and protect tumor cells against immune-mediated attack, leading to immune evasion and enhanced tumor survival.^107,108,112^ All in all, the impact of gut dysbiosis extends beyond the gastrointestinal tract and affects cancer development in a systemic manner.

### Potential clinical applications of microbiome-based therapeutics

Statement 9: Modulation of gut microbiota using probiotics or synbiotics is recommended as a complementary strategy to improve intestinal barrier functions and manage a wide array of diseases along with standard care, such as cancer, gastrointestinal, neuropsychiatric, skin, endocrine, and respiratory conditions. *(A: 37.5%; B: 50.0%; C: 12.5%; D: 0%; E: 0%).*

**Figure 4.**
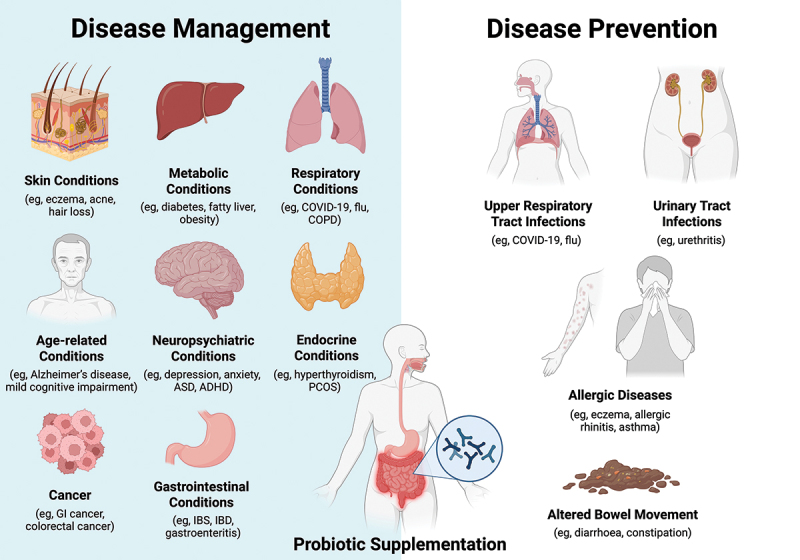
Potential applications in disease management and prevention. Supplementation of probiotics or synbiotics may offer health benefits such as altering host immune response, lowering the growth of pathogenic microorganisms and enhancing the microbial balance. For individuals with existing diseases, probiotics and synbiotics may possibly alleviate symptoms and reduce chronic inflammation. For healthy individuals, the regular use of probiotics and synbiotics may serve as a preventive, immunomodulatory measure to reduce the risk of infections. Further research is warranted to optimize these microbiome-based interventions and incorporate them into the standard treatment regimens for different diseases. Figure created with BioRender.com.

Given the robust associations between gut microbiota and disease pathogenesis as highlighted by a growing body of research, modulation of gut microbiota is a promising approach in maintaining health as well as mitigating disease progression (Figure 4).^20^ Probiotics and synbiotics have been shown by numerous studies to effectively modulate the gut microbiota composition, improve microbial diversity, strengthen intestinal barrier functions, and manage chronic inflammation.^23^ These data shed light on the potential of probiotic supplementation to serve as a complementary therapy to standard care, especially for conditions where the efficacy of conventional treatments is limited to fully manage symptoms and progression. In the context of cancer, standard treatments such as surgery, chemotherapy, and immunotherapy, while effective, commonly come with side effects such as diarrhea, gastrointestinal mucositis, and gut dysbiosis.^113^ Previous data suggested that probiotics may potentially reduce the risk of chemotherapy-induced diarrhea and mucositis in cancer patients.^114,115^ Gastrointestinal disorders, such as irritable bowel syndrome (IBS), are challenging to be managed with standard therapies. Probiotics may serve as an adjunct therapy for IBS to reduce abdominal pain and symptom severity scores.^116^ Other examples of potential indications include neuropsychiatric conditions (e.g., depression, anxiety), skin conditions (e.g., atopic dermatitis, acne vulgaris), endocrine/metabolic disorders (e.g., obesity, diabetes mellitus), and respiratory conditions (e.g., asthma, chronic obstructive pulmonary disease).^117–123^ Nevertheless, the efficacy of probiotics and synbiotics could vary depending on the use of specific microbial strains and dosages.^124^ In addition to the promising and positive effects reported across previous studies, there are also studies that have reported a lack of expected benefits for certain indications (e.g., prevention of *Clostridioides difficile* infection, maintenance of microbiome diversity following antibiotics) as shown by a few systematic reviews and meta-analyses.^125,126^ Further research is warranted to optimize these microbiome-based interventions and incorporate them into the standard treatment regimens for different diseases.

Statement 10 Supplementation of probiotics or synbiotics may offer health benefits such as altering the host immune response, lowering the growth of pathogenic microorganisms, and enhancing the microbial balance, when used in a continuous manner. Regular maintenance of gut microbial balance is recommended for both healthy individuals and those with a disease. *(A: 37.5%; B: 62.5%; C: 0%; D: 0%; E: 0%).*

Regular supplementation of probiotics or synbiotics offers multifaceted health benefits, including the promotion of digestive and overall health, immunomodulation, and the restoration of gut microbial balance.^23^ By promoting the growth of beneficial bacteria and inhibiting the growth of pathogenic bacteria, probiotics and synbiotics may help to restore and maintain a healthy microbiota.^23^ This is applicable not only to individuals suffering from diseases but also for healthy individuals seeking to maintain their health status and prevent diseases.^23,117–123,127–135^ For individuals with existing diseases, probiotics and synbiotics may possibly alleviate symptoms and reduce chronic inflammation.^23,117–123,127^ For healthy individuals, the regular use of probiotics and synbiotics may serve as a preventive, immunomodulatory measure to reduce the risk of infections, including upper respiratory tract infections (URTIs) and urinary tract infections (UTIs), as well as allergic diseases and diarrhea.^128–135^ These effects are believed to be most pronounced when the microbiome-based interventions are used continuously instead of as a one-time intervention, given the dynamically changing nature of the gut microbiota that may require regular maintenance for optimal functioning.^136,137^ Factors including antibiotic use, diet high in ultra-processed food, emulsifier, and preservatives and low in dietary fiber, may affect the growth of beneficial bacteria.^21,22,41^ Regular supplementation with probiotics or synbiotics may help to compensate for these negative influences and support a resilient and diverse gut microbiota. Short-chain fatty acids (SCFAs) produced by probiotic bacteria are known to be involved in immunomodulation through regulating the inhibition of pro-inflammatory cytokines, the promotion of anti-inflammatory cytokines, as well as the functions of immune cells.^23,101^ This immunomodulatory effect may be particularly beneficial for individuals with immune-related conditions, for instance, autoimmune conditions or allergies. In addition, synbiotic supplementation may potentially strengthen the intestinal barrier through enhancing the expression of tight junction proteins and regulating mucus production of goblet cells in the gut, to prevent the translocation of pathogenic bacteria and metabolites into the bloodstream (“leaky gut”) and the subsequent inflammatory response.^23^ Nevertheless, the potential use of probiotics and synbiotics in increasing SCFA levels and the expression of tight junction proteins remains a debatable area.^138,139^ More mechanistic and clinical research are needed to thoroughly understand the underlying mechanisms and optimize the clinical application. In the above settings, the optimal strains, dosages, and duration of supplementation require further definition, and the effects may vary according to age, health status, and the delivery matrix. While probiotics and synbiotics show promising potential for preventive health applications, their use is most effective when tailored to individual needs, taking into account the specific indications, target population, strain selection, appropriate dosing, and safety considerations, rather than using as a universal preventive measure. Additional research will help optimize these therapeutic approaches and establish more precise clinical guidelines.

Statement 11: When selecting synbiotics, the probiotic strains should be chosen based on the health benefits that it may provide to the individual as guided by both local and global scientific and clinical evidence, while prebiotics or other substrates are used in combination to promote the growth and activities of beneficial members of the gut microbiota and provide a health benefit. *(A: 37.5%; B: 62.5%; C: 0%; D: 0%; E: 0%).*

**Figure 5.**
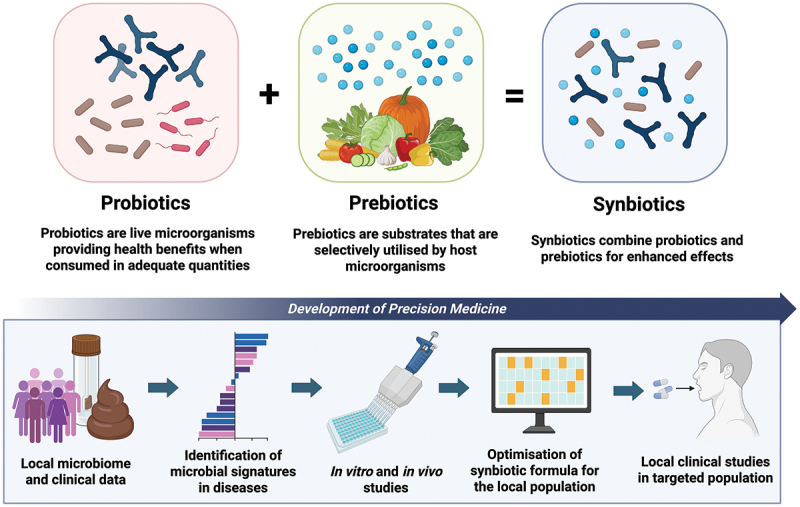
Development of microbiome-based therapeutics. Probiotics are defined as live microorganisms that, when administered in sufficient amount, confer a health benefit on the host. In addition to probiotics, prebiotics are substrates that are selectively utilized by host microorganisms conferring a health benefit. Through combining the right probiotic bacteria with the suitable prebiotic, synbiotics may offer a synergistic effect that enhances the survival, colonization, and activity of the beneficial gut bacteria, thereby offering optimal health benefits. Synbiotics that are scientifically formulated based on fecal metagenomic data represent a highly personalized and targeted approach to modulating the gut microbiome. Figure created with BioRender.com.

Probiotics are defined as live microorganisms that, when administered in sufficient amount, confer a health benefit on the host.^24^ The selection of probiotics should consider the specific health needs and medical history of an individual, as well as proven efficacy and safety in providing the desired health benefits as shown by local and global scientific and clinical evidence. Some advanced technologies for preserving the viability and ensuring colonization of live probiotics, such as microencapsulation, may also be taken into consideration to maximize the desired health benefits.^140^ Key probiotic strains that are seen in most over-the-counter formulations, including *Bifidobacterium* and *Lactobacillus*, have been extensively studied in clinical and animal studies for their health-promoting and immunomodulatory potential across various organ systems.^27,28^ Nevertheless, it is important to note that the clinical benefits of probiotics may be species-specific and strain-specific, meaning that health benefits attributed to one species or one strain may not be applicable to others, even within the same genus and species.^124^ In addition to probiotics, prebiotics are substrates that are selectively utilized by host microorganisms conferring a health benefit.^25^ They are non-digestible fibers, such as fructo-oligosaccharides (FOS), galacto-oligosaccharides (GOS), xylo-oligosaccharides (XOS), inulin, and resistant starch, which are being fermented by the gut microbiota.^25^ The selection of prebiotics should be based on its ability to support the growth of specific probiotic strains in the formulation.^25^ Through combining the right probiotic bacteria with the suitable prebiotic, synbiotics may offer a synergistic effect that enhances the survival, colonization, and activity of the beneficial gut bacteria, thereby offering optimal health benefits.^26^ Synbiotics that are scientifically formulated based on fecal metagenomic data represent a highly personalized and targeted approach to modulating the gut microbiome, when compared to “one-size-fits-all” formulations. Fecal metagenomic analysis allows the identification of microbial signatures that may be contributing to disease states.^10–12^ Through leveraging these data, the synbiotic formula could be precisely tailored to address the enriched or depleted microbial species related to diseases, enhancing the chance of achieving the desired health outcomes (Figure 5).

Statement 12 Microbial signatures of health and diseases are heterogeneous across geographical locations. It is recommended to take local clinical and microbiome data into consideration when determining the most optimal synbiotics for the local population. *(A: 100%; B: 0%; C: 0%; D: 0%; E: 0%)*

Multiple factors, including host genetics, environment, exposure to pathogens, diet and lifestyles, and cultural practices could influence the gut microbiota composition and functions.^22^ Thus, gut microbial signatures of health and diseases could exhibit significant variability across geographical locations. In particular, dietary patterns of different regions play a central role in shaping the gut microbiome.^21,22^ For instance, urban populations in developed countries may harbor gut microbiome characterized by reduced microbial diversity and increased relative abundance of microbes associated with Westernized diet and metabolic disorders.^21,22^ Such heterogeneity has crucial implications for the clinical application of probiotics and synbiotics. To optimize the efficacy of microbiome-based interventions, it is important to consider local clinical and microbiome data, as the “one-size-fits-all” approach would not address the distinct microbial and health profiles across different populations. Integrated analysis of clinical and metagenomic data is especially important in assessing the efficacy of probiotic interventions for the local population. To date, a number of local studies in Hong Kong have utilized fecal metagenomics in the characterization of local microbiome profiles, the design of targeted synbiotic formula, and the assessment of therapeutic effects.^141–144^ For instance, a synbiotic formula (SIM01) was developed locally using metagenomic analysis and identification of probiotic species that had the greatest positive correlations with the relative abundance of short-chain fatty acid (SCFA)-producing species, which are known to boost immunity and manage respiratory infections.^141,142^ Subsequent clinical studies on SIM01 showed robust therapeutic potential in the management of acute coronavirus disease 2019 (COVID-19) and post-acute COVID-19 syndrome (PACS) in the local population.^141,142^ Another synbiotic formula (SMT04) that was developed based on a local metagenomic dataset of colorectal cancer (CRC) also showed efficacy in reducing the level of gut microbial gene markers associated with CRC, including *Fusobacterium nucleatum*.^144^ Thus, this approach has a promising potential to address the unique health needs of different populations, paving the way for a new era of microbiome-based therapeutics and precision medicine.

**Statement 13**: It is a common misbelief that higher doses and more probiotic bacteria assure greater health benefits. The optimal combination of relative proportions of specific bacteria, as guided by available scientific and clinical evidence, is the most important factor governing clinical outcomes. *(A: 75.0%; B: 25.0%; C: 0%; D: 0%; E: 0%).*

The optimal dosing of probiotics and synbiotics is best achieved by adhering to evidence-based guidelines and selecting formulations that have been rigorously tested in clinical trials. The Food and Agriculture Organization (WHO) and the World Health Organization (FAO) emphasized the importance of using “adequate amount” of probiotics to achieve the desired health benefits in their definition for probiotics.^24^ The belief that higher doses and more probiotic bacteria assure greater health benefits has been a widespread misconception that overlooks the potential precision in the use of microbiome-based therapeutics. The efficacy of probiotics and synbiotics does not only depend upon the quantity of probiotic bacteria but rather by the specific probiotic strains used, the relative proportions in the formula, as well as their ability to integrate into the host microbial community to exert health benefits in a synergistical manner.^124^ The optimal combination of specific bacteria, guided by robust scientific and clinical evidence, is the most critical factor governing clinical outcomes. Current literatures, including systematic reviews and meta-analyses, do not provide clear evidence of a significant relationship between dosage and therapeutic effect.^145^ Furthermore, the misbelief that higher doses of probiotics are better also overlooks the potential side effects related to excessive consumption. While probiotics are generally considered safe, in general, cautions should be taken in critically ill, post-operative, and immunocompromised individuals.^146^ All in all, selecting probiotic strains and formulations with proven clinical efficacy and safety is far more important than the quantity of bacteria consumed.

## Conclusion

The gut microbiota play robust roles in health and disease pathogenesis through gut-organ axes and inflammatory-related pathways. Microbiome-based therapeutics, including probiotics, prebiotics, and synbiotics, may serve as a targeted and immunomodulatory approach for disease prevention and management. To our knowledge, this is the first initiative in Hong Kong involving medical experts across multiple medical specialties (i.e., gastroenterology, neurology, psychiatry, dermatology, endocrinology, geriatrics, surgery, family medicine) to discuss and advocate a set of practical guidelines for the general use of probiotics and synbiotics. Through conducting an extensive review on both global and local research findings and leveraging their combined expertise, the working group engaged in thorough discussions on the cutting-edge microbiome data and proposed potential indications requiring further research and clinical applications. The collaborative effort not only serves to inform and advance clinical practice locally but also has the potential to influence healthcare professionals in Asia and worldwide.

## Data Availability

Data sharing is not applicable to this article.
